# Regional abnormalities in the hippocampus and verbal memory impairment in craniofacial dystonia

**DOI:** 10.3389/fnins.2025.1636362

**Published:** 2025-12-17

**Authors:** Gang Liu, Huiming Liu, Linchang Zhong, Yuhan Luo, Zhengkun Yang, Jiana Zhang, Xiuye He, Zilin Ou, Weixi Zhang, Kangqiang Peng, Jinping Xu, Zhicong Yan, Yue Zhang

**Affiliations:** 1 Department of Neurology, Huidong People’s Hospital, Huizhou, China; 2 Guangdong Provincial Key Laboratory for Diagnosis and Treatment of Major Neurological Diseases, Department of Neurology, The First Affiliated Hospital, National Key Clinical Department and Key Discipline of Neurology, Sun Yat-sen University, Guangzhou, China; 3 State Key Laboratory of Oncology in Southern China, Department of Medical Imaging, Collaborative Innovation Center for Cancer Medicine, Sun Yat-sen University Cancer Center, Guangzhou, China; 4 Institute of Biomedical and Health Engineering, Shenzhen Institutes of Advanced Technology, Chinese Academy of Sciences, Shenzhen, China

**Keywords:** craniofacial dystonia, hippocampal subfields, structural magnetic resonance imaging, verbal memory, FreeSurfer

## Abstract

**Background:**

Patients with craniofacial dystonia (CFD) often present with verbal memory deficits, but their neuroanatomical basis is not yet clear. This study aims to determine whether verbal memory deficits in CFD are associated with structural atrophy of specific hippocampal subfields, and to isolate dystonia-specific pathological changes through comparison with patients with hemifacial spasm (HFS).

**Methods:**

We recruited 50 patients with CFD, 48 patients with HFS, and 50 healthy controls (HCs). Verbal memory and global cognitive function were assessed using the Rey Auditory Verbal Learning Test (RAVLT) and the Mini-Mental State Examination (MMSE), respectively. Volumes of hippocampal subfields were quantified from high-resolution T1-weighted magnetic resonance imaging (MRI) using FreeSurfer. Group comparisons were performed after controlling for relevant covariates.

**Results:**

While global cognition (MMSE) scores did not differ significantly among groups, patients with CFD demonstrated significant verbal memory deficits. Compared with HCs, they performed worse across immediate, short-, and long-delay recall trials, with medium-to-large effect sizes (all *P-FDR* ≤ 0.002; Cohen’s *d* = −0.70 to −0.82). Similar deficits of medium effect sizes were observed when compared with patients with HFS (all *P-FDR* ≤ 0.027; Cohen’s *d* = −0.49 to −0.52). Crucially, patients with HFS were unimpaired relative to HCs, establishing this memory deficit as specific to the dystonia pathophysiology. The imaging analysis revealed that patients with CFD were associated with significant atrophy in the left granule cell layer of the dentate gyrus (GC-DG) (*P-FDR* = 0.014; Cohen’s *d* = −0.58) and CA4 (*P-FDR* = 0.010; Cohen’s *d* = −0.60) compared with HCs, and with significant atrophy in the right GC-DG (*P-FDR* = 0.032; Cohen’s *d* = −0.52) and CA4 (*P-FDR* = 0.041; Cohen’s *d* = −0.50) compared with HCs. However, the magnitude of the atrophy showed no significant correlation with verbal memory scores, disease duration, or motor severity, revealing a critical structure-function dissociation.

**Conclusion:**

Our findings reveal a structure-function dissociation in CFD. We propose its verbal memory deficits, despite hippocampal atrophy, may stem from broader network dysfunction or microstructural pathology not seen on conventional MRI. This challenges models assuming a direct link between macrostructural atrophy and cognitive symptoms.

## 1 Introduction

Idiopathic adult-onset dystonia (IAOD), a common movement disorder, is characterized by motor features and non-motor manifestations. Among the non-motor manifestations, sensory and neuropsychiatric abnormalities are more commonly reported. However, deficits in cognitive function, including memory, aspects of social cognition, executive function, and changes in information processing speed, are also widely reported in patients with IAOD (Czekóová et al., 2017; Yang et al., 2017; Burke et al., 2020; Monaghan et al., 2021). Within the clinical spectrum of dystonia, increasing evidence has highlighted the link between cognitive impairment and patient quality of life (Girach et al., 2019; Ndukwe et al., 2020). However, the mechanisms underlying cognitive dysfunction in IAOD remain largely unknown.

Deficits in cognitive function in patients with dystonia were traditionally thought to be linked to basal ganglia dysfunction, as supported by evidence of impaired cognitive function in neurodegenerative disorders such as Parkinson’s, Huntington’s, or Wilson’s disease, as well as brain injury with damage to the basal ganglia (Tremblay et al., 2015; Galantucci et al., 2017; Hu et al., 2021; Puig-Davi et al., 2021) or related circuit dysfunction, including cortico-basal ganglia networks and cerebello-thalamo-cortical and cerebello-thalamo-basal ganglia circuits (Defazio et al., 2024). Indeed, as recent evidence underscores, the disruptions in the dentato-rubro-olivary pathway, which is essential for motor coordination, may represent a component of circuit dysfunction involving the cortico-basal ganglia networks and cerebello-thalamo-cortical and cerebello-thalamo-basal ganglia circuits, all of which contribute to the pathophysiology of movement disorders (Ogut et al., 2023). The basal ganglia are involved in not only prominent sensorimotor functions but also in cognitive operations and emotional-motivational processes (Rinnerthaler et al., 2006; Gan et al., 2022). Nevertheless, the neuroanatomical substrates differ among different types of IAOD (Neychev et al., 2011). Blepharospasm (BSP) may be related to structural abnormalities in motor cortices such as the supplementary motor area (Xu et al., 2023). In addition, cognitive deficits are reportedly caused by the distracting effects of motor symptoms of dystonia (Redondo-Vergé, 2001). However, a literature review reported that deep brain stimulation did not improve concurrent mild cognitive impairment (MCI) despite improved motor symptoms in patients with dystonia (Owen et al., 2015; Cernera et al., 2019). Moreover, recent studies reported a lack of association between the severity of dystonia and the severity of cognitive deficits in patients with isolated dystonia (Yang et al., 2016; Foley et al., 2017; Monaghan et al., 2021; Rafee et al., 2023; Defazio et al., 2024), providing further evidence that cognitive dysfunction is not a mere consequence of abnormal movements. Interpreting cognitive deficits in dystonia requires rigorously addressing key confounders. Pharmacological agents, particularly anticholinergics, are well-documented to have direct effects on cognitive function (Taylor et al., 1991). More significantly, the high prevalence of depression and anxiety in this population presents a major methodological challenge. Because these mood disorders are themselves associated with memory and executive dysfunctions, this creates a critical ambiguity: are the observed cognitive deficits a core feature of dystonia, or simply a byproduct of co-occurring mood symptoms (Lange et al., 2016)? While some evidence suggests these deficits persist after controlling for such factors, disentangling these intertwined variables demands a robust study design (Romano et al., 2014; Defazio et al., 2024). Therefore, a primary objective of our study was to methodologically isolate the neuroanatomical correlates of verbal memory impairments intrinsic to dystonia. To achieve this, we prospectively mitigated these confounders through stringent exclusion criteria for medications with potential cognitive side effects, and subsequently controlled for subclinical mood symptoms in our statistical models. This design permits a more direct, unconfounded examination of the link between hippocampal integrity and memory function.

Studies in recent decades have highlighted the crucial role of the medial temporal lobe (MTL), particularly the hippocampus, in memory (Cutsuridis and Wennekers, 2009; den Heijer et al., 2012; Huhn et al., 2018). Moreover, the hippocampus contains distinct subfields that serve different types of memory functions. Specifically, verbal memory is mainly associated with the anterior subfields of the left hippocampus, while visual memory is associated with many posterior subfields of the bilateral hippocampus (Travis et al., 2014). Therefore, the hippocampus should not be considered as a single homogeneous structure, as doing so may disregard potentially useful information about distinct hippocampal subfields. However, to our knowledge, no studies have explored the structural alterations in distinct hippocampal subfields and their relationship with memory alterations in patients with dystonia. A statistical atlas of the hippocampal formation was recently constructed using ultra-high-resolution *ex vivo* magnetic resonance imaging (MRI) combined with *in vivo* data (Iglesias et al., 2015). This atlas is integrated into FreeSurfer 6^1^. This tool enables the accurate measurement of each hippocampal subfield.

This study aimed to elucidate the neuroanatomical basis of verbal memory deficits in dystonia by examining hippocampal subfield integrity. Our study compared Rey Auditory Verbal Learning Test (RAVLT) scores and hippocampal subfield volumes across the three groups, and crucially, examined the links between hippocampal atrophy, memory deficits, and key clinical variables such as disease duration and severity. The present study focuses specifically on patients with craniofacial dystonia (CFD), a subtype that offers a unique clinical model for two key reasons. First, memory impairments are consistently reported as cognitive deficits in this population (Alemán et al., 2009; Yang et al., 2016; Maggi et al., 2019). Second, the clinical presentation of CFD permits a robust comparative design. By including a carefully matched control group of patients with hemifacial spasm (HFS), which is a condition with similar facial hyperkinesia but of peripheral origin, we can methodologically disentangle central dystonic pathophysiology from the confounding effects of chronic facial muscle overactivity (Dias et al., 2009).

## 2 Materials and methods

### 2.1 Participants

The patients were recruited from an outpatient clinic for movement disorders at the First Affiliated Hospital of Sun Yat-sen University. The diagnoses of HFS and CFD were made by two senior neurologists (G Liu and WX Zhang) using standard criteria (Lefaucheur et al., 2018; Defazio et al., 2019, 2021; Neuro-ophthalmology Group of Ophthalmology Branch of Chinese Medical Association et al., 2025). Specifically, patients with CFD were further classified into subtypes based on the distribution of dystonic movements: (1) Blepharospasm (BSP) is primarily manifested as stereotyped, bilateral, synchronous spasms of the orbicularis oculi muscle (leading to eyelid narrowing/closure) and an abnormally elevated blink frequency (>16 times/min), in addition to sensory symptoms such as ocular dryness, foreign body sensation, and photophobia. The characteristic sensory tricks phenomenon is also observed. Furthermore, the symptoms disappear during sleep. (2) Blepharospasm-oromandibular dystonia (BOD), building upon the clinical manifestations of BSP, involves involuntary spasmodic contractions of multiple muscle groups including the orbicularis oris, masseter, and lingual muscles. It presents as perioral twitching and abnormal mandibular movements, which may impair functions such as mastication, swallowing, and articulation. Additionally, this subtype demonstrates a tendency to spread to adjacent muscle groups. In addition, patients were excluded if they: (i) received botulinum toxin (BoNT) injections within 3 months before the MRI scan; (ii) reported evidence of stroke, traumatic brain injury, Parkinson’s disease, Alzheimer’s disease, epilepsy; (iii) had a family history of movement disorders or history of antipsychotic medication use before dystonia onset; (iv) were receiving benzodiazepines, anticholinergics, or other medications with potential cognitive side effects; (v) had any contraindications to cerebral MRI; (vi) had evidence of major psychiatric disorders. Exclusion of major psychiatric disorders was determined through a multi-component, clinician-administered assessment conducted by two senior neurologists, consistent with the principles of an integrated neuropsychiatric evaluation (Trapp et al., 2022; Pallanti, 2024). The assessment included: (a) a comprehensive neuropsychiatric history, incorporating a thorough review of medical records with an emphasis on the patient’s personal experience of events and careful screening for underlying or comorbid conditions; (b) a systematic clinical interview to screen for core symptom clusters of major mood, anxiety, and psychotic disorders; (c) a formal mental status examination assessing appearance, behavior, affect, thought process, and content; and (d) the administration of the Hamilton Depression Rating Scale (HAMD)-17 (Hamilton, 1960), the Hamilton Anxiety Rating Scale (HAMA)-14 (Maier et al., 1988), and the Mini-Mental State Examination (MMSE) (Folstein et al., 1975) to quantify symptom severity. A participant was excluded if this integrated clinical judgment, supported by direct observation, scale scores, and historical data, indicated a major psychiatric disorder that could significantly confound the study’s outcomes. Healthy controls (HCs) were recruited using the same exclusion criteria.

### 2.2 Clinical assessment

Before MRI, the participants’ demographic and clinical characteristics, including age, sex, education, disease duration, and BoNT injection duration, were collected during face-to-face interviews. The Burke-Fahn-Marsden Dystonia Rating Scale (BFMDRS) (Burke et al., 1985) and Cohen’s Scale (Cohen et al., 1986) were used to assess the severity of CFD and HFS, respectively. HAMA and HAMD were used to assess anxiety and depression symptoms, respectively.

### 2.3 Cognitive assessments

Cognitive function was evaluated using the MMSE for general cognition and the Chinese version of the Rey Auditory Verbal Learning Test (RAVLT) for verbal memory (Tsai et al., 2015). All assessments were administered in Mandarin Chinese by a single trained neurologist (Z.L. Ou) in a quiet, distraction-free environment. Standardized instructions were followed according to the respective test manuals, and all delay intervals for the RAVLT were strictly timed. From the RAVLT, we derived several key verbal memory metrics: immediate recall (total score across trials 1-3), short-delay recall, long-delay recall, cued recall, and recognition. Additionally, we calculated the short-delay forgetting rate (SDFR) and long-delay forgetting rate (LDFR), based on the formulas: SDFR = (short-delay recall score - trial 3 score)/trial 3 score, and LDFR = (long-delay recall score - trial 3 score)/trial 3 score. The RAVLT is a well-established measure with good reliability and validity for assessing verbal memory (Pliskin et al., 2021).

### 2.4 MRI data acquisition

Three-dimensional T1-weighted data were collected using a 3T MRI scanner (Tim Trio; Siemens, Erlangen, Germany) with a magnetization-prepared rapid-acquisition gradient-echo pulse sequence. The main parameters were as follows: repetition time, 2,530 ms; echo time, 4.45 ms; inversion time, 1,100 ms; flip angle, 7°; matrix dimensions, 256 × 256; voxel size, 1 × 1 × 1 mm^3^; and 192 slices.

### 2.5 Data pre-processing

**FIGURE 1:**
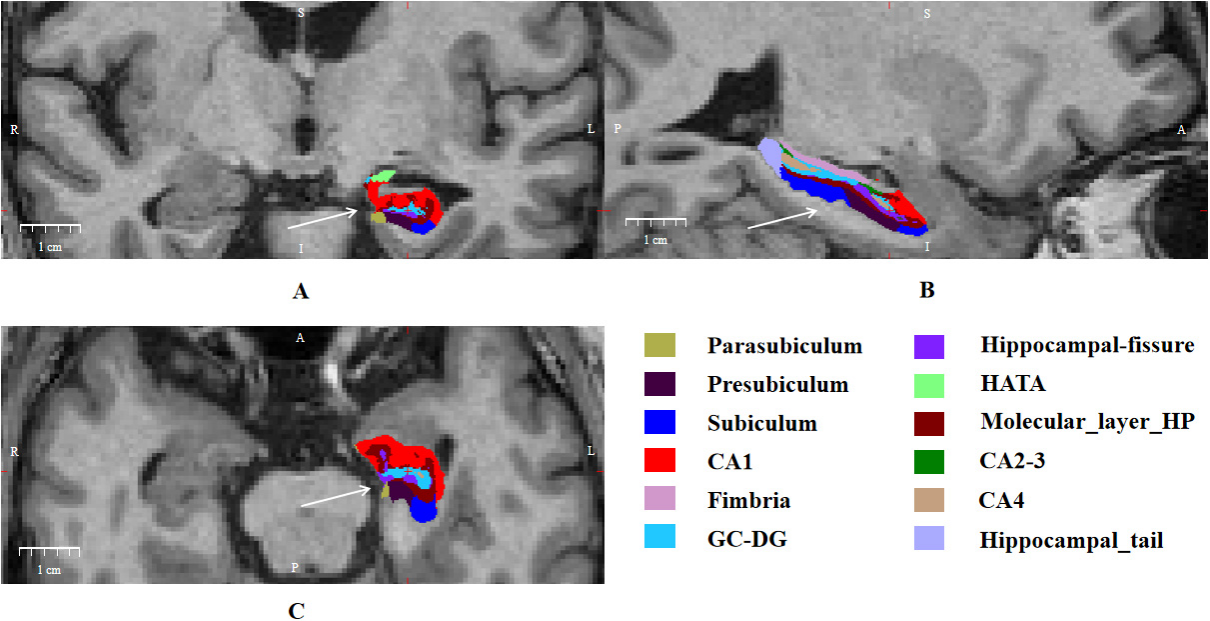
Hippocampal subfield segmentation in a patient with dystonia. **(A)** A coronal view of the left hippocampus. **(B)** A sagittal view showing the longitudinal extent of the left hippocampus. **(C)** An axial view of the left hippocampus. White arrows indicate the segmented hippocampal formation. Anatomical directions are marked on each panel (L, Left; R, Right; A, Anterior; P, Posterior; S, Superior; I, Inferior). The color legend defines the different hippocampal subfields. CA, cornu ammonis; GC-DG, granule cell layer of dentate gyrus; HATA, hippocampus-amygdala transition area; HP, hippocampus. Scale bar = 1 cm.

All T1 images were processed using the standard segmentation pipeline available in FreeSurfer 6.0. The technical details have been described elsewhere (Fischl and Dale, 2000). We used the command for volumetric segmentation (“recon-all”). The main steps included motion correction, skull stripping, intensity normalization, automated Talairach transformation, gray or white matter tessellation, and topology correction (Fischl et al., 2001; Ségonne et al., 2007). The subcortical structures were segmented using a non-linear warping atlas (Fischl et al., 2002). The total hippocampal volumes were obtained and estimated total intracranial volume (eTIV) was calculated. Subsequently, the hippocampal subfields were segmented using a Bayesian inference approach and a novel atlas algorithm for hippocampal formations built primarily on ultra-high-resolution *ex vivo* MRI data from autopsy brains (Iglesias et al., 2015). The segmentation accurately delineated various hippocampus subfields in each hemisphere, including the parasubiculum, presubiculum, subiculum, cornu ammonis (CA) 1, CA2-3, CA4, granule cell layer of dentate gyrus (GC-DG), hippocampus-amygdala transition area (HATA), fimbriae, molecular layer hippocampus (HP), hippocampal fissure, and hippocampal tail (Figure 1). Finally, Freeview^2^ was used to visualize the hippocampal subfields. We selected FreeSurfer 6.0 as it was the stable, validated, and widely adopted standard at the study’s outset. First, it introduced a novel module for hippocampal subfield segmentation based on an ultra-high-resolution *ex vivo* atlas (Iglesias et al., 2015). Second, its excellent test-retest reliability has been well-documented in large-scale studies (Haddad et al., 2023). Third, given that different software versions can yield systematic volumetric differences affecting statistical outcomes (Sämann et al., 2022; Haddad et al., 2023), we used this single, consistent pipeline for all processing. This approach ensures both the internal consistency of our dataset and its comparability with the substantial body of literature using this well-characterized tool.

### 2.6 Volume calculations in the hippocampal subfields

The volumes of the bilateral hippocampal subfields were calculated and intergroup comparisons were performed using analysis of covariance (ANCOVA), with age, sex, education, eTIV, HAMA, and HAMD scores included as covariates. The statistical method was specifically chosen to isolate the effect of group by statistically controlling for the influence of these potential confounders. By accounting for variance associated with the covariates, ANCOVA increases statistical power and provides a more precise and less biased estimate of the adjusted group means (Porter and Raudenbush, 1987; Egbewale et al., 2014). Moreover, these comparisons were all corrected for multiple testing using the False Discovery Rate (FDR) method with the Benjamini-Hochberg procedure. Statistical significance was set at *P-FDR* < 0.05.

### 2.7 Correlation analyses

To explore potential confounding effects of clinical variables within the CFD group, we conducted partial correlation analyses to examine the relationships between (1) RAVLT memory scores and hippocampal subfield volumes, (2) RAVLT memory scores and clinical variables (duration and disease severity), and (3) hippocampal subfield volumes and clinical variables (duration and disease severity). All partial correlation analyses controlled for age, sex, HAMA, and HAMD scores as covariates. Given the exploratory nature of these analyses, we applied FDR correction for multiple comparisons. Statistical significance was set at *P-FDR* < 0.05.

### 2.8 Statistical analyses

Differences in demographic variables among the three groups were assessed based on their distribution. For normally distributed data, one-way analysis of variance (ANOVA) was used, while the non-parametric Kruskal-Wallis *H*-test was employed for variables such as age and educational level, which did not meet the assumption of normality. Sex comparisons were performed using chi-square (χ^2^) tests. ANCOVA was used to assess intergroup differences in RAVLT scores. Following a significant main effect in the omnibus ANCOVA, planned *post hoc* pairwise comparisons were conducted using the FDR method. Statistical significance was set at *P-FDR* < 0.05. These analyses were performed using IBM SPSS Statistics for Windows, version 26.0 (IBM Corp., Armonk, NY, United States).

## 3 Results

### 3.1 Participant characteristics and behavioral assessment

This study enrolled 50 patients with CFD (40 with BSP and 10 with BOD), 48 patients with HFS, and 50 HCs. The demographic information, clinical characteristics, and behavioral test scores of all participants are detailed in Table 1 and the details of ANCOVA results for RAVLT scores among three groups are presented in Table 2. Age, sex, education, or MMSE scores did not differ significantly among the three groups. Patients with CFD displayed significantly lower performance in measures of immediate recall, short-delay recall, and long-delay recall compared with the patients with HFS (all *P-FDR* ≤ 0.027; Cohen’s *d* = −0.49 to −0.52), and HCs (all *P-FDR* ≤ 0.002; Cohen’s *d* = −0.70 to −0.82). All reported *P*-*FDR* values were corrected for multiple comparisons using the FDR method. Moreover, patients with HFS did not show evidence of deterioration in these measures relative to HCs. In addition, cued recall, SDFR, LDFR, and recognition did not differ significantly among the groups.

**TABLE 1:** Participant characteristics and behavioral assessment.

Characteristics	CFD (*n* = 50)	HFS (*n* = 48)	HCs (*n* = 50)	*P*	CFD < HFS	CFD < HCs	HFS < HCs
Subjects (BSP/BOD)	40/10	–	–	–	–	–	–
Median age, y (range)	54 (30–68)	52 (30–69)	52 (29–65)	0.698^b^	–	–	–
Median education, y (range)	10.5 (0–16)	12 (0–16)	12 (0–16)	0.399^b^	–	–	–
Sex (female/male)	29/21	29/19	29/21	0.906^a^	–	–	–
eTIV, mm^3^ (range)	1489590.1 (921606.1–1968076.7)	1442859.7 (1082096.8–1960182.6)	1499080.0 (1088275.0–1819172.5)	0.872^c^	–	–	–
Median MMSE scores (range)	27 (24–30)	27 (24–30)	28 (24–30)	0.418^b^	–	–	–
Median disease duration, y (range)	2 (0.170–15)	2 (0.080–20)	–	0.195^d^	–	–	–
Median Cohen scores (range)	–	3 (1–4)	–	-	–	–	–
Median BFMDRS scores (range)	4.5 (3–10.5)	–	–	–	–	–	–
BoNT injections (yes/no)	31/19	27/21	–	0.07^a^	–	–	–
Median BoNT injection duration, y (range)	0 (0-15)	0 (0-19)	–	0.958^d^	–	–	–
Median HAMA scores (range)	4.5 (0–14)	3 (0–9)	2 (0–10)	**0.002^b^**	**0.031**	**0.002**	0.105
Median HAMD scores (range)	4 (0–12)	2 (0–10)	1 (0–13)	**0.001^b^**	**0.005**	**0.001**	0.444
**RAVLT^e^**							
Median immediate recall (range)	12 (4–26)	14.5 (9–25)	17 (8–26)	**<0.001**	**0.022**	**<0.001**	0.059
Median short-delay recall (range)	4 (0–9)	5 (2–10)	5 (0–11)	**0.002**	**0.018**	**0.002**	0.278
Median long-delay recall (range)	3 (0–8)	4 (0–10)	5 (0–10)	**0.001**	**0.027**	**<0.001**	0.125
Median SDFR (range)	−0.293 (−1.000 to 1.000)	−0.236 (−0.667 to 0.600)	−0.211 (−1.000 to 0.250)	0.580	–	–	–
Median LDFR (range)	−0.400 (−1.000 to 1.000)	−0.317 (−1.000 to 0.500)	−0.236 (−1.000 to 0.125)	0.880	–	–	–
Median cued recall (range)	3 (0–10)	4 (0–8)	5 (0–10)	0.287	–	–	–
Median recognition (range)	20 (15–24)	20 (15–24)	21 (11–24)	0.963	–	–	–

^a^Represents χ^2^ test.

^b^Represents Kruskal-Wallis *H*-test between three groups.

^c^Represents one-way analysis of variance, and subsequent *post hoc* analyses between any two groups.

^d^Represents Mann-Whitney U test between two groups.

^e^Represents ANCOVA between three groups on each RAVLT sub-score with age, sex, education, HAMA, and HAMD scores as covariates and the results were corrected for multiple comparisons using False Discovery Rate. Statistically significant differences (*P* < 0.05) are highlighted in bold. ANCOVA, analysis of covariance; BFMDRS, Burke-Fahn-Marsden Dystonia Rating Scale; BOD, blepharospasm oromandibular dystonia; BoNT, Botulinum toxin; BSP, blepharospasm; CFD, craniofacial dystonia; eTIV, estimated total intracranial volume; HAMA, the Hamilton Rating Scale for Anxiety; HAMD, the Hamilton Rating Scale for Depression; HCs, healthy controls; HFS, hemifacial spasm; LDFR, long-delay forgetting rate; MMSE, Mini-Mental State Examination; RAVLT, Rey Auditory Verbal Learning Test; SDFR, short-delay forgetting rate; y, years.

**TABLE 2:** ANCOVA results for RAVLT scores.

RAVLT measures	*F*	*P*	$\eta_{P}^{2}$	CFD vs. HFS MD (95% CI) *P-FDR*, Cohen’s *d*	CFD vs. HCs MD (95% CI) *P-FDR*, Cohen’s *d*	HFS vs. HCs MD (95% CI) *P-FDR*, Cohen’s *d*
Immediate recall	5.33	<0.001	0.071	−2.23 (−4.02, −0.45) **0.022**, *d* = −0.50	−3.94 (−5.88, −2.00) **<0.001**, *d* = −0.81	−1.70 (−3.48, 0.07) 0.059, *d* = −0.39
Short-delay recall	3.17	0.002	0.043	−1.07 (−1.89, −0.24) **0.018**, *d* = −0.52	−1.58 (−2.48, −0.68) **0.002**, *d* = -0.70	−0.51 (−1.45, 0.42) 0.278, *d* = −0.22
Long-delay recall	4.54	0.001	0.061	−1.04 (−1.90, −0.18) **0.027**, *d* = −0.49	−1.80 (−2.67, −0.93) **<0.001**, *d* = −0.82	−0.76 (−1.73, 0.21) 0.125, *d* = −0.31
SDFR	0.55	0.58	0.008	–	–	–
LDFR	0.13	0.88	0.002	–	–	–
Cued recall	1.26	0.287	0.018	–	–	–
Recognition	0.04	0.963	0.001	–	–	–

The results shown are from a series of ANCOVA used to compare the three groups on each RAVLT sub-score with age, sex, education, HAMA, and HAMD scores as covariates. The results were corrected for multiple comparisons using FDR. ηP2, partial eta-squared. Statistically significant differences (*P* < 0.05) are highlighted in bold. ANCOVA, analysis of covariance; CFD, craniofacial dystonia; CI, confidence interval; FDR, False Discovery Rate; HAMA, the Hamilton Rating Scale for Anxiety; HAMD, the Hamilton Rating Scale for Depression; HCs, healthy controls; HFS, hemifacial spasm; LDFR, long-delay forgetting rate; MD, mean difference; RAVLT, Rey Auditory Verbal Learning Test; SDFR, short-delay forgetting rate.

### 3.2 Hippocampal segmentation

**FIGURE 2:**
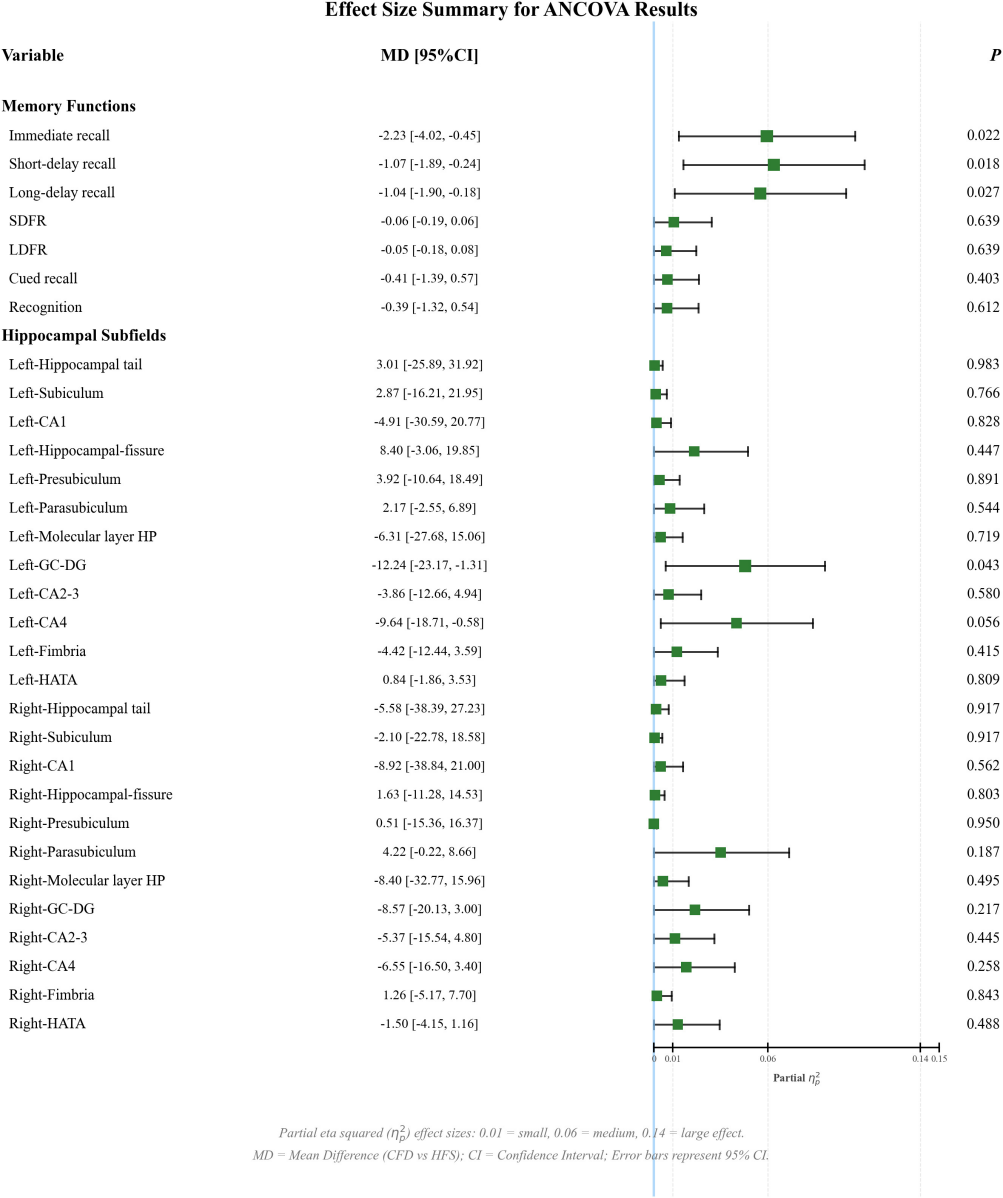
ANCOVA results of verbal memory and hippocampal subfields (CFD vs. HFS). The forest plot displays adjusted mean difference and 95% confidence interval. *P*-values are FDR-corrected. Thresholds for partial eta-squared (η_*p*_^2^) effect sizes are shown. Models controlled for age, sex, education, HAMA, and HAMD (eTIV for subfield volumes). ANCOVA, analysis of covariance; CA, cornu ammonis; CFD, craniofacial dystonia; eTIV, estimated total intracranial volume; GC-DG, granule cell layer of the dentate gyrus; HAMA, the Hamilton Rating Scale for Anxiety; HAMD, the Hamilton Rating Scale for Depression; HATA, hippocampus-amygdala transition area; HFS, hemifacial spasm; LDFR, long-delay forgetting rate; SDFR, short-delay forgetting rate.

**FIGURE 3:**
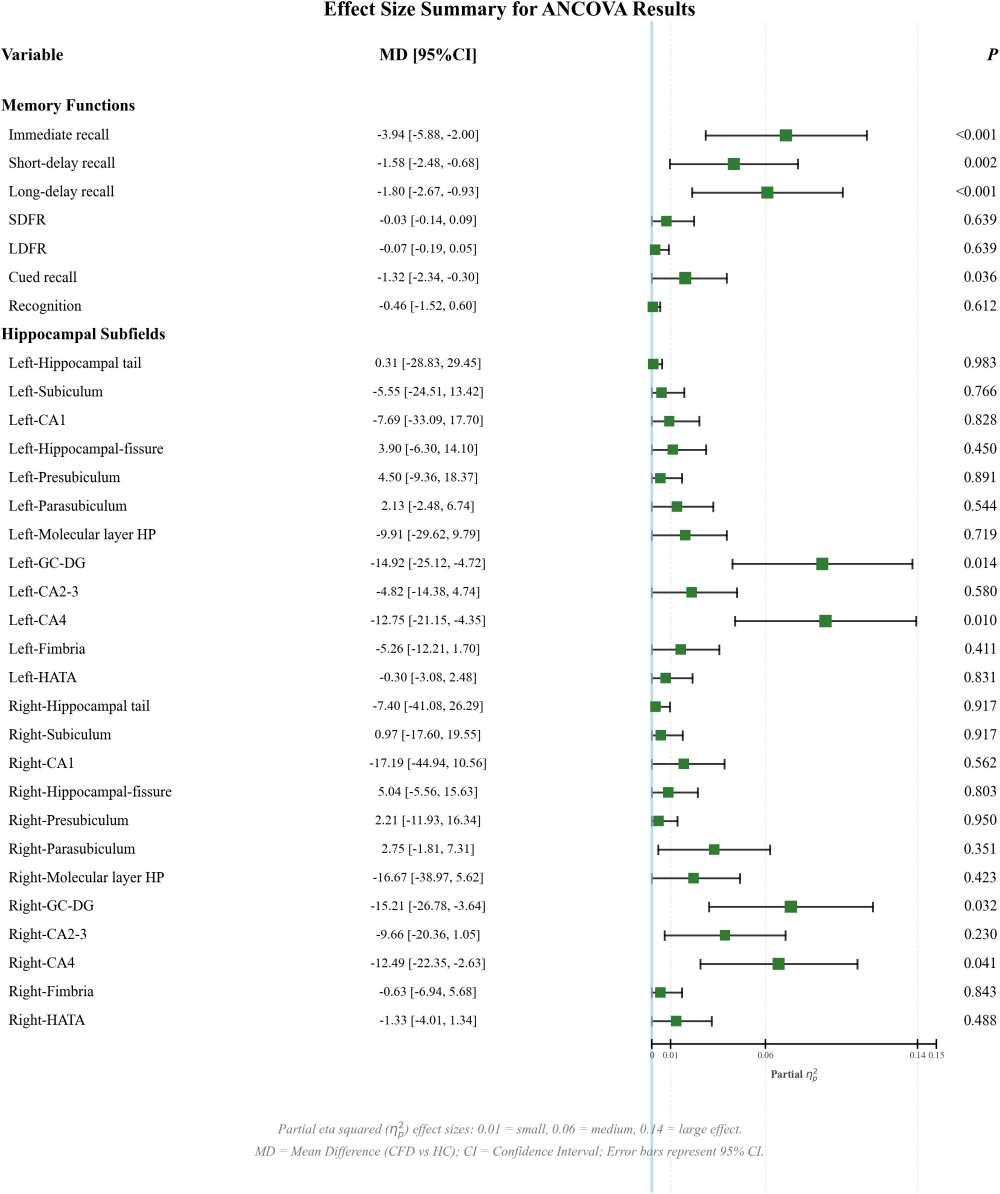
ANCOVA results of verbal memory and hippocampal volumes (CFD vs. HCs). The forest plot displays adjusted mean differences and 95% confidence interval. *P*-values are FDR-corrected. Thresholds for partial eta-squared (η_*p*_^2^) effect sizes are shown. Models controlled for age, sex, education, HAMA, and HAMD (eTIV for subfield volumes). ANCOVA, analysis of covariance; CA, cornu ammonis; CFD, craniofacial dystonia; eTIV, estimated total intracranial volume; GC-DG, granule cell layer of the dentate gyrus; HAMA, the Hamilton Rating Scale for Anxiety; HAMD, the Hamilton Rating Scale for Depression; HATA, hippocampus-amygdala transition area; HCs, healthy controls; LDFR, long-delay forgetting rate; SDFR, short-delay forgetting rate.

After controlling for sex, age, education, eTIV, HAMA, and HAMD scores, the imaging analysis revealed that CFD was associated with significant atrophy in the left GC-DG (*P-FDR* = 0.014; adjusted mean difference (MD) = −14.92; 95% confidence interval (CI) = −25.12 to −4.72; Cohen’s *d* = −0.58; reduction 5.13%) and CA4 (*P-FDR* = 0.010; adjusted MD = −12.75; 95% CI = −21.15 to −4.35; Cohen’s *d* = −0.60; reduction 5.11%) compared with HCs, with significant atrophy in the left GC-DG (*P-FDR* = 0.043; adjusted MD = −12.24; Cohen’s *d* = −0.45; reduction 4.23%), and with significant atrophy in the right GC-DG (*P-FDR* = 0.032; adjusted MD = −15.21; 95% CI = −26.78 to −3.64; Cohen’s *d* = −0.50; reduction 5.05%) and CA4 (*P-FDR* = 0.041; adjusted MD = −12.49; 95% CI = −22.35 to −2.63; Cohen’s *d* = −0.50; reduction 4.87%) compared with HCs (Tables 3, 4). All reported *P-FDR* values were corrected for multiple comparisons using the FDR method. Forest plots visualizing the adjusted MD, 95% CI for all key comparisons are presented in Figure 2 (CFD vs. HFS) and Figure 3 (CFD vs. HCs).

**TABLE 3:** Volumetric data of hippocampal subfields.

Hippocampal subfields	CFD (mean ± SD)	HFS (mean ± SD)	HCs (mean ± SD)	*P*	CFD < HFS	CFD < HCs	HFS < HCs
**Left**							
Parasubiculum	61.328 ± 12.449	59.154 ± 11.011	59.201 ± 10.721	0.394	–	–	–
Presubiculum	320.889 ± 35.300	316.965 ± 37.330	316.385 ± 34.580	0.724	–	–	–
Subiculum	442.200 ± 48.928	439.333 ± 46.112	447.750 ± 46.610	0.697	–	–	–
CA1	598.621 ± 64.763	603.535 ± 63.242	606.312 ± 63.195	0.525	–	–	–
CA2-3	184.786 ± 21.143	188.644 ± 22.735	189.608 ± 26.704	0.228	–	–	–
CA4	236.582 ± 20.297	246.227 ± 24.791	249.329 ± 21.992	**0.001**	0.056	**0.010**	0.513
GC-DG	275.724 ± 25.240	287.964 ± 29.179	290.641 ± 26.158	**0.001**	**0.043**	**0.014**	0.633
HATA	56.249 ± 7.333	55.414 ± 6.009	56.549 ± 6.672	0.809	–	–	–
Fimbria	80.850 ± 18.434	85.271 ± 21.481	86.106 ± 16.560	0.343	–	–	–
Molecular_layer_HP	548.375 ± 53.394	554.685 ± 53.148	558.287 ± 45.594	0.290	–	–	–
Hippocampal-fissure	154.936 ± 28.204	146.540 ± 28.923	151.036 ± 22.912	0.459	–	–	–
Hippocampal_tail	569.500 ± 69.409	566.490 ± 74.745	569.200 ± 77.226	0.948	–	–	–
**Right**							
Parasubiculum	56.811 ± 12.315	52.594 ± 9.589	54.061 ± 10.580	0.098	–	–	–
Presubiculum	306.591 ± 37.610	306.084 ± 41.495	304.382 ± 33.502	0.778	–	–	–
Subiculum	455.142 ± 50.922	457.239 ± 52.207	454.169 ± 42.280	0.716	–	–	–
CA1	631.283 ± 74.170	640.200 ± 75.034	648.471 ± 65.378	0.306	–	–	–
CA2-3	195.765 ± 24.668	201.140 ± 26.051	205.422 ± 29.110	0.065	–	–	–
CA4	244.250 ± 23.787	250.801 ± 25.841	256.740 ± 25.859	**0.008**	0.258	**0.041**	0.258
GC-DG	285.670 ± 28.283	294.238 ± 29.400	300.880 ± 30.017	**0.005**	0.217	**0.032**	0.271
HATA	55.411 ± 6.806	56.906 ± 6.419	56.744 ± 6.676	0.405	–	–	–
Fimbria	76.917 ± 14.990	75.652 ± 17.065	77.547 ± 16.759	0.725	–	–	–
Molecular_layer_HP	568.537 ± 60.176	576.942 ± 61.311	585.210 ± 51.860	0.211	–	–	–
Hippocampal-fissure	160.431 ± 29.158	158.803 ± 35.048	155.395 ± 23.976	0.543	–	–	–
Hippocampal_tail	591.478 ± 80.276	597.056 ± 83.348	598.874 ± 89.243	0.869	–	–	–

Differences of hippocampal subfields volume among three groups were analyzed using analysis of covariance with age, sex, education, eTIV, HAMA, and HAMD scores as covariates. The results were corrected using False Discovery Rate method. Statistically significant differences (*P* < 0.05) are highlighted in bold. CA, cornu ammonis; CFD, craniofacial dystonia; eTIV, estimated total intracranial volume; GC-DG, granule cell layer of dentate gyrus; HAMA, Hamilton Rating Scale for Anxiety; HAMD, Hamilton Rating Scale for Depression; HATA, hippocampus-amygdala transition area; HCs, healthy controls; HFS, hemifacial spasm; HP, hippocampus.

**TABLE 4:** ANCOVA results for volume of the hippocampal subregions.

Hippocampal subfields	*F*	*P*	ηP2	CFD vs. HFS MD (95%CI) *P-FDR*, Cohen’s *d*	CFD vs. HCs MD (95%CI) *P-FDR*, Cohen’s *d*	HFS vs. HCs MD (95%CI) *P-FDR*, Cohen’s *d*
**Left**						
Parasubiculum	0.94	0.394	0.013	–	–	–
Presubiculum	0.32	0.724	0.005	–	–	–
Subiculum	0.36	0.697	0.005	–	–	–
CA1	0.65	0.525	0.009	–	–	–
CA2-3	1.49	0.228	0.021	–	–	–
CA4	7.01	**0.001**	0.092	−9.64 (−18.71, −0.58) 0.056, *d* = −0.43	−12.75 (−21.15, −4.35) **0.010**, *d* = −0.60	−3.10 (−12.49, 6.29) 0.513, *d* = −0.13
GC-DG	6.87	**0.001**	0.090	−12.24 (−23.17, −1.31) **0.043**, *d* = −0.45	−14.92 (−25.12, −4.72) **0.014**, *d* = −0.58	−2.68 (13.78, 8.43) 0.633, *d* = −0.10
HATA	0.51	0.601	0.007	–	–	–
Fimbria	1.08	0.343	0.015	–	–	–
Molecular_layer_HP	1.25	0.290	0.018	–	–	–
Hippocampal-fissure	0.78	0.459	0.011	–	–	–
Hippocampal_tail	0.05	0.947	0.001	–	–	–
Right						
Parasubiculum	2.36	0.098	0.033	–	–	–
Presubiculum	0.25	0.778	0.004	–	–	–
Subiculum	0.34	0.716	0.005	–	–	–
CA1	1.19	0.306	0.017	–	–	–
CA2-3	2.79	0.065	0.039	–	–	–
CA4	4.98	**0.008**	0.067	−6.55 (−16.50, 3.40) 0.258, *d* = −0.26	−12.49 (−22.35, −2.63) **0.041**, *d* = −0.50	−5.94 (−16.31, 4.43) 0.258, *d* = −0.23
GC-DG	5.50	**0.005**	0.073	−8.57 (−20.13, 3.00) 0.217, *d* = −0.30	−15.21 (−26.78, −3.64) **0.032**, *d* = −0.52	−6.64 (−18.56, 5.28) 0.271, *d* = −0.22
HATA	0.91	0.405	0.013	–	–	–
Fimbria	0.32	0.725	0.005	–	–	–
Molecular_layer_HP	1.57	0.212	0.022	–	–	–
Hippocampal-fissure	0.61	0.543	0.009	–	–	–
Hippocampal_tail	0.14	0.869	0.002	–	–	–

The results shown are from a series of ANCOVA used to compare the three groups on volume of the hippocampal subregions with age, sex, education, eTIV, HAMA, and HAMD scores as covariates. The results were corrected for multiple comparisons using FDR. ηP2, partial eta-squared. Statistically significant differences (*P* < 0.05) are highlighted in bold. ANCOVA, analysis of covariance; CA, cornu ammonis; CFD, craniofacial dystonia; CI, confidence interval; eTIV, estimate total intracranial volume; FDR, False Discovery Rate; GC-DG, granule cell layer of dentate gyrus; HAMA, the Hamilton Rating Scale for Anxiety; HAMD, the Hamilton Rating Scale for Depression; HATA, hippocampus-amygdala transition area; HCs, healthy controls; HFS, hemifacial spasm; HP, hippocampus; MD, mean difference.

### 3.3 Correlation analyses

Within the CFD group, we performed partial correlation analyses to investigate potential associations between cognitive performance, hippocampal subfield volumes, and clinical characteristics, while controlling for age, sex, HAMA, and HAMD scores. These analyses specifically examined the relationships between: (1) RAVLT memory scores and hippocampal subfield volumes; (2) RAVLT memory scores and clinical variables (disease duration and BFMDRS scores); and (3) hippocampal subfield volumes and clinical variables (disease duration and BFMDRS scores). After applying FDR correction across all comparisons, none of these correlations reached statistical significance (all *P-FDR* > 0.05) (Table 5).

**TABLE 5:** Partial correlation analyses of hippocampal volumes, memory scores, and clinical variables in patients with CFD.

Variable 1	Variable 2	*r*	*P-FDR*
**Memory scores vs. Hippocampal subfield volumes**			
Immediate recall	Left GC-DG	−0.147	0.798
	Left CA4	−0.124	0.921
	Right GC-DG	0.018	0.971
	Right CA4	0.041	0.971
Short-delay recall	Left GC-DG	−0.005	0.971
	Left CA4	0.015	0.971
	Right GC-DG	0.011	0.971
	Right CA4	−0.007	0.971
Long-delay recall	Left GC-DG	0.013	0.971
	Left CA4	0.018	0.971
	Right GC-DG	0.097	0.971
	Right CA4	0.057	0.971
**Memory scores vs. Clinical variables**			
Immediate recall	Disease duration	0.153	0.798
	BFMDRS	0.008	0.971
Short-delay recall	Disease duration	0.164	0.798
	BFMDRS	0.054	0.971
Long-delay recall	Disease duration	0.203	0.798
	BFMDRS	0.150	0.798
**Hippocampal subfield volumes vs. clinical variables**			
Left GC-DG	Disease duration	0.063	0.971
	BFMDRS	0.373	0.146
Right GC-DG	Disease duration	0.022	0.971
	BFMDRS	0.329	0.171
Left CA4	Disease duration	0.163	0.798
	BFMDRS	0.356	0.146
Right CA4	Disease duration	0.033	0.971
	BFMDRS	0.291	0.263

The results shown are from a series of partial correlation used to compare the variables with age, sex, HAMA, and HAMD scores as covariates. For every comparison, this table provides the partial correlation coefficient (*r*) and the precise *P-FDR* value, corrected for multiple comparisons using the Benjam-Hochberg FDR procedure. BFMDRS, Burke-Fahn-Marsden Dystonia Rating Scale; CFD, craniofacial dystonia; FDR, False Discovery Rate; HAMA, the Hamilton Rating Scale for Anxiety; HAMD, the Hamilton Rating Scale for Depression.

## 4 Discussion

The present study provides compelling evidence for a specific verbal memory impairment in patients with CFD, which persists even when compared to a clinical control group with similar hyperkinetic movements. This cognitive deficit appears to be an intrinsic feature of the disorder, as it showed no correlation with motor severity or disease duration. Concurrently, we identified significant bilateral atrophy in the GC-DG and CA4 hippocampal subfields. The pivotal finding of our study, however, is the striking dissociation between these structural and functional alterations: the extent of this bilateral atrophy was not associated with the severity of verbal memory impairment. This lack of a direct relationship is particularly paradoxical, as more extensive, bilateral damage to memory-related structures would logically be expected to correlate with functional decline. These results firmly establish altered cognitive function as an integral part of the CFD clinical spectrum and suggest a complex, non-linear pathophysiology that cannot be explained by macroscopic atrophy alone. Future longitudinal research with larger sample sizes is essential to unravel the mechanisms underlying this structure-function dissociation.

In this study, while MMSE scores did not differ significantly between patients with CFD and the HCs, the immediate recall, short-delay recall, and long-delay recall scores were significantly lower in patients with CFD compared with the HCs, suggesting that although the overall cognitive function may appear to be normal; certain specific cognitive domains such as verbal memory are impaired in patients with CFD. Impairment of memory function has been reported using other neuropsychological batteries in primary blepharospasm and cranial-cervical dystonia (Romano et al., 2014). Such subtle cognitive alterations may be associated with the distracting effects of abnormal movements, as suggested by improvements in attention impairment after BoNT treatment in patients with cervical dystonia (Allam et al., 2007). The excessive movement in patients with CFD likely did not affect their scores on the verbal memory tests because these patients performed significantly worse on the RAVLT when compared with patients with HFS, consistent with findings of previous literature demonstrating impairments in cognitive flexibility in patients with BSP compared with patients with hyperkinetic symptoms of non-dystonic origin (Lange et al., 2016). The absence of a significant association between symptom severity in patients with CFD and verbal memory deficits, including immediate, short- and long-delay recall, also supports this theory. These findings suggest that the altered verbal memory function in patients with CFD results from specific pathophysiological processes underlying dystonia.

A key finding of the present study is the observed dissociation between hippocampal subfield atrophy and the severity of verbal memory deficits in patients with CFD. This paradox is underscored by our discovery of bilateral atrophy in the GC-DG and CA4 subfields. Given that these subfields, particularly within the left hemisphere, are widely regarded as critical neural substrates for verbal memory (Maguire, 2001; Ezzati et al., 2016; Tsalouchidou et al., 2023), one would logically anticipate that more extensive, bilateral structural damage would manifest in a clear structure-function relationship. Instead, the complete absence of such a correlation directly challenges the conventional assumption of a linear link between macroscopic atrophy and cognitive impairment in dystonia. This observation suggests that conventional etiological models may be insufficient, pointing to the potential value of a more comprehensive framework to better understand the pathophysiology of cognitive deficits in CFD. However, before proposing novel biological frameworks, it is imperative to critically evaluate the methodological factors that could potentially account for this null finding.

Several methodological factors warrant consideration as potential explanations for the lack of a significant correlation. First, statistical power limitations are a well-documented challenge in clinical neuroscience research. As seminal work highlights, underpowered studies not only have a reduced chance of detecting a true effect but also decrease the likelihood that a statistically significant result reflects a true effect (Button et al., 2013). While our study identified significant group differences, the sample size may have been insufficient to detect a potentially subtle covariance between subfield atrophy and cognitive scores, risking a Type II error. Second, the technical challenges of hippocampal subfields segmentation must be acknowledged. Automated segmentation relies on standardized protocols and atlases, which, while robust, can be susceptible to inaccuracies, especially in brains with existing atrophy or anatomical variability (Goubran et al., 2020). Such measurement variance could obscure a true, underlying structure-function relationship. Third, the cross-sectional nature of our study limits causal inference. It is plausible that atrophy and cognitive decline follow different temporal trajectories. These methodological considerations demand caution in interpreting the null finding as definitive biological dissociation. Finally, we did not separately analyze the BSP and BOD subgroups. Given the imbalanced sample sizes in our cohort (40 patients with BSP vs. 10 patients with BOD), a formal statistical comparison would have been severely underpowered, and its results could be unreliable or misleading. Future studies with larger, more balanced cohorts are warranted to investigate potential subtype-specific effects. Nevertheless, while these limitations warrant caution, the consistency of our primary findings encourages a deeper exploration of the potential biological mechanisms underlying this dissociation.

Assuming the observed dissociation is not solely a methodological artifact but reflects a true biological phenomenon, we propose that the primary driver of verbal memory deficits in CFD may not originate from focal hippocampal pathology, but from dysfunction within large-scale cortico-basal ganglia networks. Dystonia is increasingly understood not as a disorder of the basal ganglia alone, but as a brain-wide network disorder (Gill et al., 2023). Foundational work has established that these circuits, subserving both motor and non-motor functions, are critically implicated in the pathophysiology of dystonia (Alexander et al., 1986; Stamelou et al., 2012; Ray et al., 2020). Crucially, cognitive and psychiatric features are now recognized as core components of the “non-motor syndrome” of dystonia (Stamelou et al., 2012). Verbal memory impairment, as observed here, may be attributable to disruptions in frontally-mediated executive strategies—such as inefficient encoding and impaired strategic retrieval—which are direct consequences of basal ganglia pathophysiology (Monaghan et al., 2021). This perspective is further supported by evidence that chronic dysregulation within cortico-striatal circuits can induce remote structural changes in connected regions like the hippocampus. Such processes may operate through mechanisms of altered trophic support (Brito et al., 2014). In this “network-first” view, the focal hippocampal atrophy we observed may represent a downstream consequence of chronic network dysregulation or a bystander marker of a global disease process, rather than the causal driver of the cognitive symptoms.

Secondly, the dissociation between macroscopic structure and function may reflect pathological processes occurring at a scale not captured by conventional volumetric MRI. Although we did not find a significant correlation between atrophy in the bilateral GC-DG and CA4 subfields and delayed recall performance in CFD patients, previous experimental animal studies have shown that hippocampal subregions such as CA1 and CA3 are highly vulnerable to oxidative damage, suggesting that structural and molecular disruptions in these areas—whether from toxic exposure or underlying disease—can impair memory-related processes (Ogut et al., 2019). This is highly relevant, as a growing body of evidence implicates mitochondrial dysfunction and oxidative stress as core pathophysiological concepts in dystonia (Koptielow et al., 2025). Therefore, it is plausible that molecular disruptions (e.g., from oxidative stress) in vulnerable subfields impair memory-related processes long before they culminate in detectable atrophy. This concept of functional impairment preceding volumetric loss is powerfully illustrated in the neurodegeneration literature. In Alzheimer’s disease, cognitive decline correlates more strongly with synaptic loss—a “synaptopathy”—than with frank neuronal loss (Terry et al., 1991; Selkoe, 2002). It is therefore plausible that the verbal memory deficits in our CFD cohort reflect an underlying synaptopathy within the memory network (perhaps driven by the oxidative stress mechanisms suggested by animal models), while the volumetric changes we measured represent a delayed, less sensitive proxy.

## 5 Conclusion

This study reveals a critical apparent paradox in CFD: focal hippocampal atrophy occurs alongside significant verbal memory deficits, yet the two are not directly correlated. This finding challenge simplistic structure-function models. After rigorously considering potential methodological limitations (e.g., statistical power, segmentation accuracy), we hypothesize this dissociation may reflect two possibilities: (1) the cognitive impairment is driven by large-scale basal ganglia network dysfunction, for which hippocampal atrophy is a downstream marker; or (2) the deficits stem from underlying microstructural pathology (e.g., synaptopathy) not captured by conventional MRI. Clinically, our findings establish verbal memory impairment as a core feature of CFD that warrants assessment independently of structural MRI findings. Future longitudinal, multi-modal neuroimaging studies are necessary to test these competing network and microstructural hypotheses.

## Data Availability

The raw data supporting the conclusions of this article will be made available by the authors, without undue reservation.

## Footnotes

1
https://surfer.nmr.mgh.harvard.edu/fswiki/rel6downloads

2
https://surfer.nmr.mgh.harvard.edu/fswiki/FreeviewGuide/FreeviewIntro duction

## References

[B1] Alemán G. de Erausquin G. Micheli F. (2009). Cognitive disturbances in primary blepharospasm. *Mov. Disord.* 24 2112–2120. 10.1002/mds.22736 19705473

[B2] Alexander G. DeLong M. Strick P. (1986). Parallel organization of functionally segregated circuits linking basal ganglia and cortex. *Annu. Rev. Neurosci.* 9 357–381. 10.1146/annurev.ne.09.030186.002041 3085570

[B3] Allam N. Frank J. Pereira C. Tomaz C. (2007). Sustained attention in cranial dystonia patients treated with botulinum toxin. *Acta Neurol. Scand.* 116 196–200. 10.1111/j.1600-0404.2007.00862.x 17714334

[B4] Brito V. Giralt A. Enriquez-Barreto L. Puigdellívol M. Suelves N. Zamora-Moratalla A. (2014). Neurotrophin receptor p75(NTR) mediates Huntington’s disease-associated synaptic and memory dysfunction. *J. Clin. Invest.* 124 4411–4428. 10.1172/JCI74809 25180603 PMC4191006

[B5] Burke R. Fahn S. Marsden C. Bressman S. Moskowitz C. Friedman J. (1985). Validity and reliability of a rating scale for the primary torsion dystonias. *Neurology* 35 73–77. 10.1212/wnl.35.1.73 3966004

[B6] Burke T. Monaghan R. McCormack D. Cogley C. Pinto-Grau M. O’Connor S. (2020). Social cognition in cervical dystonia: A case-control study. *Clin. Park. Relat. Disord.* 3:100072. 10.1016/j.prdoa.2020.100072 34316651 PMC8298799

[B7] Button K. Ioannidis J. Mokrysz C. Nosek B. Flint J. Robinson E. (2013). Power failure: Why small sample size undermines the reliability of neuroscience. *Nat. Rev. Neurosci.* 14 365–376. 10.1038/nrn3475 23571845

[B8] Cernera S. Okun M. Gunduz A. (2019). A review of cognitive outcomes across movement disorder patients undergoing deep brain stimulation. *Front. Neurol.* 10:419. 10.3389/fneur.2019.00419 31133956 PMC6514131

[B9] Cohen D. Savino P. Stern M. Hurtig H. (1986). Botulinum injection therapy for blepharospasm: A review and report of 75 patients. *Clin. Neuropharmacol.* 9 415–429. 10.1097/00002826-198610000-00002 3533250

[B10] Cutsuridis V. Wennekers T. (2009). Hippocampus, microcircuits and associative memory. *Neural Netw*. 22, 1120–1128. 10.1016/j.neunet.2009.07.009 19647982

[B11] Czekóová K. Zemánková P. Shaw D. Bareš M. (2017). Social cognition and idiopathic isolated cervical dystonia. *J. Neural Transm.* 124 1097–1104. 10.1007/s00702-017-1725-8 28444457

[B12] Defazio G. Albanese A. Pellicciari R. Scaglione C. Esposito M. Morgante F. (2019). Expert recommendations for diagnosing cervical, oromandibular, and limb dystonia. *Neurol. Sci.* 40 89–95. 10.1007/s10072-018-3586-9 30269178

[B13] Defazio G. Jinnah H. Berardelli A. Perlmutter J. Berkmen G. Berman B. (2021). Diagnostic criteria for blepharospasm: A multicenter international study. *Parkinsonism. Relat. Disord.* 91 109–114. 10.1016/j.parkreldis.2021.09.004 34583301 PMC9048224

[B14] Defazio G. Muroni A. Taurisano P. Gigante A. Fanzecco M. Martino D. (2024). Are cognitive symptoms part of the phenotypic spectrum of idiopathic adult-onset dystonia? Summary of evidence from controlled studies. *Mov. Disord. Clin. Pract.* 11 329–334. 10.1002/mdc3.13978 38314659 PMC10982590

[B15] Den Heijer T. Der Lijn F. Vernooij M. W. De Groot M. Koudstaal P. J. Van Der Lugt A. (2012). Structural and diffusion MRI measures of the hippocampus and memory performance. *Neuroimage* 63, 1782–1789. 10.1016/j.neuroimage.2012.08.067 22960084

[B16] Dias F. Doyle F. Kummer A. Cardoso F. Caramelli P. Teixeira A. (2009). Executive functioning in patients with blepharospasm in comparison with patients with hemifacial spasm. *Arq. Neuropsiquiatr.* 67 12–15. 10.1590/s0004-282x2009000100004 19330202

[B17] Egbewale B. Lewis M. Sim J. (2014). Bias, precision and statistical power of analysis of covariance in the analysis of randomized trials with baseline imbalance: A simulation study. *BMC Med. Res. Methodol.* 14:49. 10.1186/1471-2288-14-49 24712304 PMC3986434

[B18] Ezzati A. Katz M. Zammit A. Lipton M. Zimmerman M. Sliwinski M. (2016). Differential association of left and right hippocampal volumes with verbal episodic and spatial memory in older adults. *Neuropsychologia* 93 380–385. 10.1016/j.neuropsychologia.2016.08.016 27542320 PMC5154822

[B19] Fischl B. Dale A. (2000). Measuring the thickness of the human cerebral cortex from magnetic resonance images. *Proc. Natl. Acad. Sci. U S A.* 97 11050–11055. 10.1073/pnas.200033797 10984517 PMC27146

[B20] Fischl B. Liu A. Dale A. (2001). Automated manifold surgery: Constructing geometrically accurate and topologically correct models of the human cerebral cortex. *IEEE Trans. Med. Imaging* 20 70–80. 10.1109/42.906426 11293693

[B21] Fischl B. Salat D. Busa E. Albert M. Dieterich M. Haselgrove C. (2002). Whole brain segmentation: Automated labeling of neuroanatomical structures in the human brain. *Neuron* 33 341–355. 10.1016/s0896-6273(02)00569-x 11832223

[B22] Foley J. Vinke R. Limousin P. Cipolotti L. (2017). Relationship of cognitive function to motor symptoms and mood disorders in patients with isolated dystonia. *Cogn. Behav. Neurol.* 30 16–22. 10.1097/WNN.0000000000000117 28323682

[B23] Folstein M. Folstein S. McHugh P. (1975). Mini-mental state. A practical method for grading the cognitive state of patients for the clinician. *J. Psychiatr. Res.* 12 189–198. 10.1016/0022-3956(75)90026-6 1202204

[B24] Galantucci S. Agosta F. Stefanova E. Basaia S. van den Heuvel M. Stojković T. (2017). Structural brain connectome and cognitive impairment in Parkinson disease. *Radiology* 283 515–525. 10.1148/radiol.2016160274 27924721

[B25] Gan X. Zhou X. Li J. Jiao G. Jiang X. Biswal B. (2022). Common and distinct neurofunctional representations of core and social disgust in the brain: Coordinate-based and network meta-analyses. *Neurosci. Biobehav. Rev.* 135:104553. 10.1016/j.neubiorev.2022.104553 35122784

[B26] Gill J. Nguyen M. Hull M. van der Heijden M. Nguyen K. Thomas S. (2023). Function and dysfunction of the dystonia network: An exploration of neural circuits that underlie the acquired and isolated dystonias. *Dystonia* 2:11805. 10.3389/dyst.2023.11805 38273865 PMC10810232

[B27] Girach A. Vinagre Aragon A. Zis P. (2019). Quality of life in idiopathic dystonia: A systematic review. *J. Neurol.* 266 2897–2906. 10.1007/s00415-018-9119-x 30460447 PMC6851210

[B28] Goubran M. Ntiri E. Akhavein H. Holmes M. Nestor S. Ramirez J. (2020). Hippocampal segmentation for brains with extensive atrophy using three-dimensional convolutional neural networks. *Hum. Brain Mapp.* 41 291–308. 10.1002/hbm.24811 31609046 PMC7267905

[B29] Haddad E. Pizzagalli F. Zhu A. Bhatt R. Islam T. Ba Gari I. (2023). Multisite test-retest reliability and compatibility of brain metrics derived from FreeSurfer versions 7.1, 6.0, and 5.3. *Hum. Brain Mapp.* 44 1515–1532. 10.1002/hbm.26147 36437735 PMC9921222

[B30] Hamilton M. (1960). A rating scale for depression. *J. Neurol. Neurosurg. Psychiatry* 23 56–62. 10.1136/jnnp.23.1.56 14399272 PMC495331

[B31] Hu S. Xu C. Dong T. Wu H. Wang Y. Wang A. (2021). Structural and functional changes are related to cognitive status in Wilson’s disease. *Front. Hum. Neurosci.* 15:610947. 10.3389/fnhum.2021.610947 33716691 PMC7947794

[B32] Huhn S. Beyer F. Zhang R. Lampe L. Grothe J. Kratzsch J. (2018). Effects of resveratrol on memory performance, Hippocampus connectivity and microstructure in older adults - A randomized controlled trial. *Neuroimage* 174 177–190. 10.1016/j.neuroimage.2018.03.023 29548848

[B33] Iglesias J. Augustinack J. Nguyen K. Player C. Player A. Wright M. (2015). A computational atlas of the hippocampal formation using ex vivo, ultra-high resolution MRI: Application to adaptive segmentation of in vivo MRI. *Neuroimage* 115 117–137. 10.1016/j.neuroimage.2015.04.042 25936807 PMC4461537

[B34] Koptielow J. Szyłak E. Koptielowa A. Sarnowska M. Kapica-Topczewska K. Adamska-Patruno E. (2025). Dystonia versus redox balance: A preliminary assessment of oxidative stress in patients. *Antioxidants* 14:1052. 10.3390/antiox14091052 41008959 PMC12466378

[B35] Lange F. Seer C. Dengler R. Dressler D. Kopp B. (2016). Cognitive flexibility in primary dystonia. *J. Int. Neuropsychol. Soc.* 22 662–670. 10.1017/S135561771600045X 27333537

[B36] Lefaucheur J. Ben Daamer N. Sangla S. Le Guerinel C. (2018). Diagnosis of primary hemifacial spasm. *Neurochirurgie* 64 82–86. 10.1016/j.neuchi.2017.12.003 29673578

[B37] Maggi G. D’Iorio A. Mautone G. Peluso S. Manganelli F. Dubbioso R. (2019). Cognitive correlates of prospective memory in dystonia. *Parkinsonism Relat. Disord.* 66 51–55. 10.1016/j.parkreldis.2019.06.027 31279634

[B38] Maguire E. (2001). Neuroimaging, memory and the human hippocampus. *Rev. Neurol.* 157 791–794. 11677399

[B39] Maier W. Buller R. Philipp M. Heuser I. (1988). The hamilton anxiety scale: Reliability, validity and sensitivity to change in anxiety and depressive disorders. *J. Affect. Disord.* 14 61–68. 10.1016/0165-0327(88)90072-9 2963053

[B40] Monaghan R. Cogley C. Burke T. McCormack D. O’Riordan S. Ndukwe I. (2021). Non-motor features of cervical dystonia: Cognition, social cognition, psychological distress and quality of life. *Clin. Park. Relat. Disord.* 4:100084. 10.1016/j.prdoa.2020.100084 34316662 PMC8299967

[B41] Ndukwe I. O’Riordan S. Walsh C. Hutchinson M. (2020). Trust the patient not the doctor: The determinants of quality of life in cervical dystonia. *Front. Neurol.* 11:991. 10.3389/fneur.2020.00991 33013654 PMC7499056

[B42] Neuro-ophthalmology Group of Ophthalmology Branch of Chinese Medical Association, Neuro-ophthalmology Society, and Chinese Research Hospital Association. (2025). Chinese expert consensus on the diagnosis and treatment of Meige syndrome. *Chin. J. Ocul. Fundus Dis.* 41 418–427. 10.3760/cma.j.cn511434-20250414-001693

[B43] Neychev V. Gross R. Lehéricy S. Hess E. Jinnah H. (2011). The functional neuroanatomy of dystonia. *Neurobiol. Dis.* 42 185–201. 10.1016/j.nbd.2011.01.026 21303695 PMC3478782

[B44] Ogut E. Armagan K. Tufekci D. (2023). The guillain-mollaret triangle: A key player in motor coordination and control with implications for neurological disorders. *Neurosurg. Rev.* 46:181. 10.1007/s10143-023-02086-1 37468768

[B45] Ogut E. Sekerci R. Akcay G. Yildirim F. Derin N. Aslan M. (2019). Protective effects of syringic acid on neurobehavioral deficits and hippocampal tissue damages induced by sub-chronic deltamethrin exposure. *Neurotoxicol. Teratol.* 76:106839. 10.1016/j.ntt.2019.106839 31644947

[B46] Owen T. Gimeno H. Selway R. Lin J. (2015). Cognitive function in children with primary dystonia before and after deep brain stimulation. *Eur. J. Paediatr. Neurol.* 19 48–55. 10.1016/j.ejpn.2014.09.004 25457508

[B47] Pallanti S. (2024). The role of neurosciences in clinical interviewing. *Psychother. Psychosom.* 93 282–284. 10.1159/000539165 38880086

[B48] Pliskin J. DeDios Stern S. Resch Z. Saladino K. Ovsiew G. Carter D. (2021). Comparing the psychometric properties of eight embedded performance validity tests in the rey auditory verbal learning test, wechsler memory scale logical memory, and brief visuospatial memory test-revised recognition trials for detecting invalid neuropsychological test performance. *Assessment* 28 1871–1881. 10.1177/1073191120929093 32484371

[B49] Porter A. C. Raudenbush S. (1987). Analysis of covariance: Its model and use in psychological research. *J. Couns. Psychol.* 34 383–392. 10.1037/0022-0167.34.4.383

[B50] Puig-Davi A. Martinez-Horta S. Sampedro F. Horta-Barba A. Perez-Perez J. Campolongo A. (2021). Cognitive and affective empathy in Huntington’s disease. *J. Huntingtons Dis.* 10 323–334. 10.3233/JHD-210469 34486985

[B51] Rafee S. Monaghan R. McCormack D. Fearon C. O’Riordan S. Hutchinson M. (2023). Social cognition deficits are associated with lower quality of life in cervical dystonia: A single centre study. *Clin. Park. Relat. Disord.* 9:100214. 10.1016/j.prdoa.2023.100214 39802882 PMC11724324

[B52] Ray S. Pal P. Yadav R. (2020). Non-motor symptoms in cervical dystonia: A review. *Ann. Indian Acad. Neurol.* 23 449–457. 10.4103/aian.AIAN_27_20 33223660 PMC7657286

[B53] Redondo-Vergé L. (2001). [Cognitive deterioration in Huntington disease]. *Rev. Neurol.* 32 82–85. 11293108

[B54] Rinnerthaler M. Benecke C. Bartha L. Entner T. Poewe W. Mueller J. (2006). Facial recognition in primary focal dystonia. *Mov. Disord.* 21 78–82. 10.1002/mds.20659 16114021

[B55] Romano R. Bertolino A. Gigante A. Martino D. Livrea P. Defazio G. (2014). Impaired cognitive functions in adult-onset primary cranial cervical dystonia. *Parkinsonism Relat. Disord.* 20 162–165. 10.1016/j.parkreldis.2013.10.008 24161376

[B56] Sämann P. Iglesias J. Gutman B. Grotegerd D. Leenings R. Flint C. (2022). FreeSurfer-based segmentation of hippocampal subfields: A review of methods and applications, with a novel quality control procedure for ENIGMA studies and other collaborative efforts. *Hum. Brain Mapp.* 43 207–233. 10.1002/hbm.25326 33368865 PMC8805696

[B57] Ségonne F. Pacheco J. Fischl B. (2007). Geometrically accurate topology-correction of cortical surfaces using nonseparating loops. *IEEE Trans. Med. Imaging* 26 518–529. 10.1109/TMI.2006.887364 17427739

[B58] Selkoe D. (2002). Alzheimer’s disease is a synaptic failure. *Science* 298 789–791. 10.1126/science.1074069 12399581

[B59] Stamelou M. Edwards M. Hallett M. Bhatia K. (2012). The non-motor syndrome of primary dystonia: Clinical and pathophysiological implications. *Brain* 135 1668–1681. 10.1093/brain/awr224 21933808 PMC3359748

[B60] Taylor A. Lang A. Saint-Cyr J. Riley D. Ranawaya R. (1991). Cognitive processes in idiopathic dystonia treated with high-dose anticholinergic therapy: Implications for treatment strategies. *Clin. Neuropharmacol.* 14 62–77. 10.1097/00002826-199102000-00005 2029694

[B61] Terry R. Masliah E. Salmon D. Butters N. DeTeresa R. Hill R. (1991). Physical basis of cognitive alterations in Alzheimer’s disease: Synapse loss is the major correlate of cognitive impairment. *Ann. Neurol.* 30 572–580. 10.1002/ana.410300410 1789684

[B62] Trapp N. Martyna M. Siddiqi S. Bajestan S. (2022). The neuropsychiatric approach to the assessment of patients in neurology. *Semin. Neurol.* 42 88–106. 10.1055/s-0042-1745741 35477181 PMC9177704

[B63] Travis S. Huang Y. Fujiwara E. Radomski A. Olsen F. Carter R. (2014). High field structural MRI reveals specific episodic memory correlates in the subfields of the hippocampus. *Neuropsychologia* 53 233–245. 10.1016/j.neuropsychologia.2013.11.016 24296251

[B64] Tremblay L. Worbe Y. Thobois S. Sgambato-Faure V. Féger J. (2015). Selective dysfunction of basal ganglia subterritories: From movement to behavioral disorders. *Mov. Disord.* 30 1155–1170. 10.1002/mds.26199 25772380

[B65] Tsai R. Leong J. Dutt S. Chang C. Lee A. Chao S. (2015). The Chinese verbal learning test specifically assesses hippocampal state. *Am. J. Alzheimers Dis. Other Dement.* 30 412–416. 10.1177/1533317514552667 25270640 PMC4379122

[B66] Tsalouchidou P. Müller C. Belke M. Zahnert F. Menzler K. Trinka E. (2023). Verbal memory depends on structural hippocampal subfield volume. *Front. Neurol.* 14:1209941. 10.3389/fneur.2023.1209941 37900611 PMC10613087

[B67] Xu J. Luo Y. Peng K. Guo Y. Zhong L. Liu Y. (2023). Supplementary motor area driving changes of structural brain network in blepharospasm. *Brain* 146 1542–1553. 10.1093/brain/awac341 36130317

[B68] Yang J. Shao N. Song W. Wei Q. Ou R. Wu Y. (2017). Nonmotor symptoms in primary adult-onset cervical dystonia and blepharospasm. *Brain Behav.* 7:e00592. 10.1002/brb3.592 28239516 PMC5318359

[B69] Yang J. Song W. Wei Q. Ou R. Cao B. Liu W. (2016). Screening for cognitive impairments in primary blepharospasm. *PLoS One* 11:e0160867. 10.1371/journal.pone.0160867 27526026 PMC4985064

